# Tumor-on-a-chip: Perfusable vascular incorporation brings new approach to tumor metastasis research and drug development

**DOI:** 10.3389/fbioe.2022.1057913

**Published:** 2022-11-22

**Authors:** Ruixin Wang, Chenghao Zhang, Danxue Li, Yang Yao

**Affiliations:** State Key Laboratory of Oral Diseases, National Clinical Research Center for Oral Diseases, West China Hospital of Stomatology, Sichuan University, Chengdu, China

**Keywords:** tumor-on-a-chip, vascularization, tumor model, tumor micro environment, microfluidic

## Abstract

The extracellular matrix interacts with cancer cells and is a key factor in the development of cancer. Traditional two-dimensional models cannot mimic the natural *in situ* environment of cancer tissues, whereas three-dimensional (3D) models such as spherical culture, bioprinting, and microfluidic approaches can achieve *in vitro* reproduction of certain structures and components of the tumor microenvironment, including simulation of the hypoxic environment of tumor tissue. However, the lack of a perfusable vascular network is a limitation of most 3D models. Solid tumor growth and metastasis require angiogenesis, and tumor models with microvascular networks have been developed to better understand underlying mechanisms. Tumor-on-a-chip technology combines the advantages of microfluidics and 3D cell culture technology for the simulation of tumor tissue complexity and characteristics. In this review, we summarize progress in constructing tumor-on-a-chip models with efficiently perfused vascular networks. We also discuss the applications of tumor-on-a-chip technology to studying the tumor microenvironment and drug development. Finally, we describe the creation of several common tumor models based on this technology to provide a deeper understanding and new insights into the design of vascularized cancer models. We believe that the tumor-on-a-chip approach is an important development that will provide further contributions to the field.

## Introduction

Cancer has been the second leading cause of mortality worldwide since 2018. In 2020, the global incidence of cancer was estimated to be 19.3 million new cases, with approximately 10.0 million cancer-related deaths (Ferlay et al., 2021). In China, which has a population of 1.4 billion, increases in the incidence and mortality of cancer have led to great challenges and a significant burden on the economy (Xia et al., 2022).

Much work has been devoted to understanding how cancer develops, spreads, and progresses. Classical two-dimensional (2D) cultures have been widely used over the past several decades owing to their simplicity and low cost. In these cultures, cells are plated on rigid materials and allowed to grow and proliferate in a simplified environment (Hoarau-Véchot et al., 2018). Such 2D cultures enable modification of the cellular morphology and architecture as well as cell–cell signal transmission (Yamada and Cukierman, 2007). However, 2D monolayers lack stroma, which is crucial for simulating structural architecture. The lack of matrix also leads to a lack of extracellular matrix (ECM) interaction, which is known to change the behavior of cancer cells *via* dysregulation of cell signaling. Therefore, 2D cultures cannot accurately represent a tumor under real conditions *in vivo* (Atat et al., 2022).

The local tumor microenvironment (TME), where the tumor is present, is composed of ECM, cellular components, blood vessels, and various signaling molecules (Arneth, 2019). The interactions between tumor cells and other cells in the TME can affect the development and progression of cancer (Yoon et al., 2010). The vascular network is a key factor in the TME. When the volume of a progressive solid tumor exceeds 2 mm^3^, the center of the tumor cannot receive sufficient oxygen and nutrients from the blood supply, and the cells easily become hypoxic and necrotic (Folkman, 1971; Pugh and Ratcliffe, 2003). Tumor cells in a hypoxic environment secrete angiogenic growth factors and cytokines, enabling formation of new blood vessels to meet the demands of rapid tumor growth and progression to a more malignant state (Arauchi et al., 2015). Tumor tissue also releases large quantities of cancer cells into the blood vessels and lymph vessels, enabling the cancer to spread to distant areas of the body; this process, termed metastasis, occurs in the later stages of cancer and is a leading cause of cancer-related death (Chaffer and Weinberg, 2011).

For a better understanding of the mechanisms of tumorigenesis, progression, and metastasis of tumors and the bioactivities between cells in the TME, appropriate complex tumor culture models are required. In three-dimensional (3D) cultures, tumor cells can interact with the ECM in all dimensions while maintaining their morphology, polarization, gene expression, and heterogeneity (Tibbitt and Anseth, 2009; Ridky et al., 2010; McCaffrey and Macara, 2011; Gill and West, 2014). Furthermore, the response to therapeutics is less sensitive in 3D tumor models, making them more appropriate for use in human clinical trials (Lai et al., 2011; Chwalek et al., 2014). It has been demonstrated that 3D cellular morphology is essential for tumor development, metastasis, and gene expression. Thus, 3D cultures can more precisely represent the environment of a tumor *in vivo* owing to the form and interactions of the cells.

Currently, various types of 3D models are widely used in cancer research. The cells can be grown on scaffolds using hydrogels, natural matrix, or biocompatible synthetic polymers constructed by 3D printing techniques. Alternatively, the cells can be cultured in spheroids by methods including the hanging drop technique, bioreactors, and organoids. Existing 3D tumor models have enabled *in vitro* simulation of the ECM (Hughes et al., 2021; Mosquera et al., 2022), and some progress has been made in vascularization using different models. Shoval et al. (2017) prepared spheroids by mixing cancer cells of different origins with endothelial cells in different ratios, hoping to obtain 3D tumor models with vascular networks. However, clear capillary networks were only observed in the case of patient-derived spheroids. In the human pancreatic cell line BxPC3, endothelial cells were not distributed throughout the microstructure but tended to accumulate at the periphery. A vascular network prepared in this way does not have a perfusion function. Chen et al. (2020) constructed 3D scaffolds and inoculated activated stromal cells mixed with tumor cells onto the scaffolds to achieve vascularized 3D tumor models. After 1 week, they observed the formation of structures resembling vascular lumen. However, their drug-resistance experiments continued to be performed in static culture, although the 3D model exhibited greater drug resistance.

**FIGURE 1:**
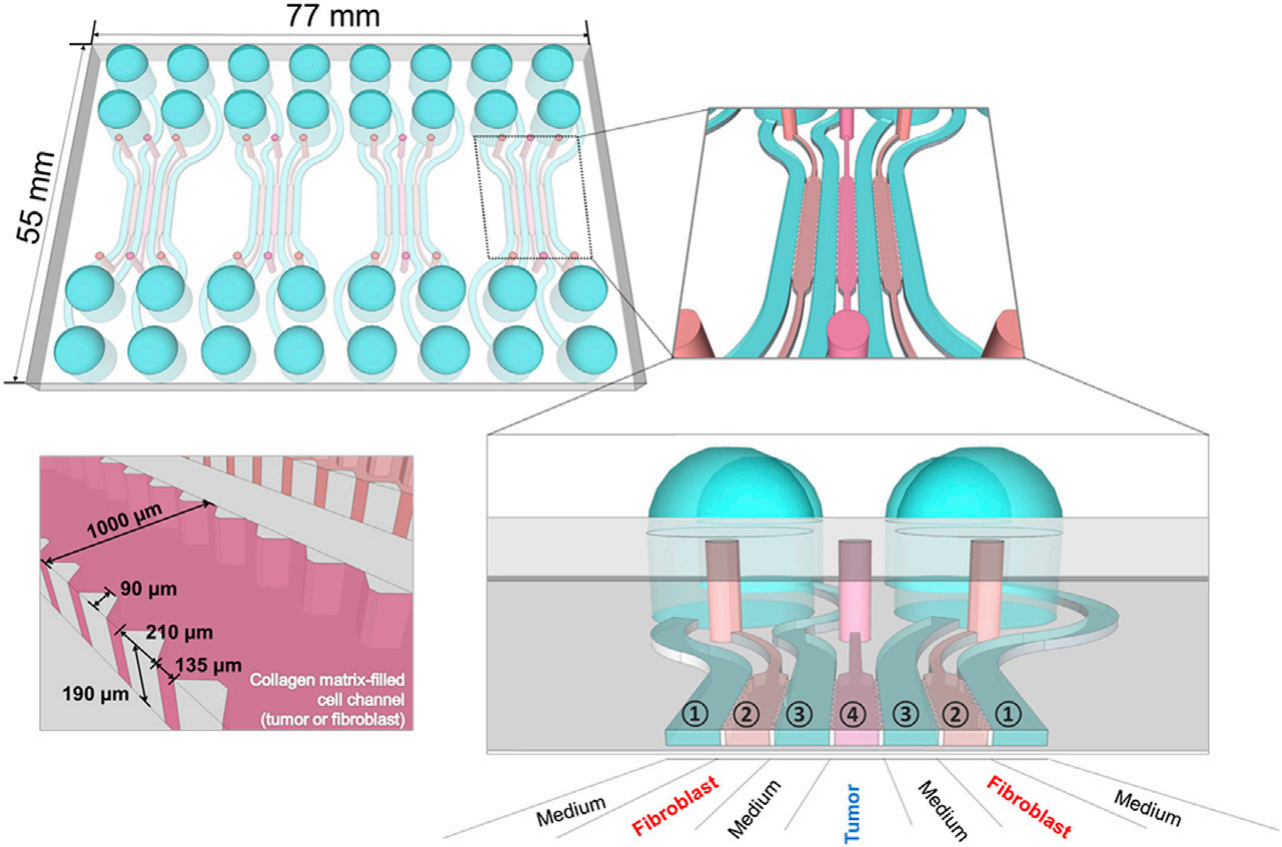
Tumor-on-a-chip consists of channels that can be loaded with media and cells and can support perfusion culture. Reprinted under the terms of the Creative Commons Attribution License (CC BY). (https://creativecommons.org/licenses/by/4.0/). Copyright 2016 Jeong et al. (2016).

In terms of functional vascularization, tumor-on-a-chip systems exhibit unique advantages. A tumor-on-a-chip is a collection of 3D tumor models and a microfluidic system that ensures continuous infusion of fresh culture fluid and simulates physiological processes such as vascular perfusion and material transport. 3D tumor models are usually developed by first using a common technique to create 3D *in vitro* tumor models, which are transferred to chips. By designing the 3D structure of tumor-on-a-chip platforms using soft lithography, 3D printing, and other technologies, complex tissue structures composed of tumor cells, stromal cells, and blood vessels can be realized (Tsai et al., 2017). Commonly used techniques include construction of scaffolds, formation of tumor spheroids, and bioink 3D printing. In microfluidics, which originated from the merging of biology and microelectronics, cells can be cultured in micrometer-sized chambers and perfused with very small amounts of fluid (Whitesides, 2006). The fluid can be injected and expelled from microchannels that are engraved on the device and linked to the macroenvironment (Unger et al., 2000). Therefore, the TME can be controlled and manipulated accurately for the analysis of cell kinetics and diffusion effects of live cells. The channels and chambers in tumor-on-a-chip devices are filled with hydrogels containing cells or perfused with fluid to mimic the *in vivo* structure and forces applied to the tumor (Poornima et al., 2022). Figure 1 shows the basic structure of a tumor-on-a-chip model. Several microfluidic devices have been used for studying cancer cell phenotypes, metastasis, the TME, vascularization, and so on, as well as for drug screening (Hou et al., 2009; Shin et al., 2011; Gioiella et al., 2016; Mannino et al., 2017; Phan et al., 2017; Nagaraju et al., 2018; Dhiman et al., 2019; Nashimoto et al., 2020).

Existing tumor-on-a-chip systems already enable physiological perfusion simulations, However, there are still great differences in permeability and spatial structure between the modeled perfusion channels and real tumor blood vessels. How to further realize perfusable vascularization on tumor-on-a-chip platforms to better simulate the TME is worth consideration. In this review, we summarize the progress in this field with regard to the construction of vascular networks and their integration with 3D cancer models. We also discuss the applications of tumor-on-a-chip technology in studying the TME and in drug development. Finally, we discuss the creation of several common tumor models based on this technology, hoping to deepen our understanding and provide new insights into the design of vascularized cancer models.

## Perfusion-ready vessel establishment in tumor-on-a-chip platform

**FIGURE 2:**
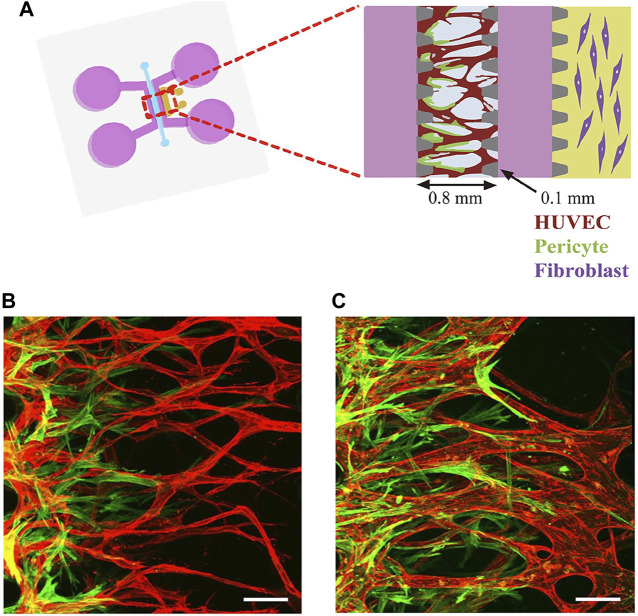
Vascularization in microfluidic devices. **(A)** Schematic diagram of the microfluidic device composed of two adjacent media channels and the outermost fibroblast channel. **(B)** third day of vascularization. **(C)** matured pericytes covered the perfusable EC network on day 6, Adapted under the terms of the Creative Commons Attribution License. Copyright Kim et al. (2015).

A tumor-on-a-chip platform, which combines a 3D model with a microfluidic device, has the great advantages of adjustable channel size and perfusion rate and can be used to study shear forces and blood flow during tumor vascularization (Haase and Kamm, 2017). In this sense, a tumor-on-a-chip platform can be considered to be similar to a tumor model with a vascular system. Further vascularization of microfluidic devices is usually achieved by inoculating endothelial cells along the tube wall. Figure 2 shows an example of vascularization in microfluidic channels.

Here, we review some common methods for vascularization of tumor-on-a-chip platforms. Some of these methods involve forming vascular channels on microfluidic devices before combining them with tumor models, whereas other methods, such as bioprinting, involve construction of vessels in 3D tumor models before connection to microfluidic channels.

### Microfabrication techniques: Photolithography and soft lithography

Photolithography is a well-known technique that has been frequently used for patterning proteins and cells. It can be used to produce patterns with features smaller than 1 μm (Park and Shuler, 2003). Kane and colleagues developed an alternative technique called soft lithography, using elastomeric materials for pattern transfer (Kane et al., 1999). In this technique, micropatterns are made using light, a photoresist, and a mask. A mask with the desired opaque pattern is made; then, the photoresist is exposed to ultraviolet light through this mask. When the exposed area of the photoresist is dissolved in a developer solution, a photoresist pattern is created. Polydimethylsiloxane (PDMS) is an ideal material for the construction of microfluidic devices using soft lithography and photolithography, owing to its transparency, flexibility, economy, ease of molding, biocompatibility, and gas permeability. It can also be used to form microfluidic devices after bonding to substrates such as glass or silicon (Borók et al., 2021).

Many studies have demonstrated that it is possible to prepare microfluidic platforms using soft lithography and successfully construct perfusable vascular networks (Zheng et al., 2012; Jeon et al., 2014; Yue et al., 2021). Fabricating cellular compartments in a vascularized microfluidic platform and introducing tumor tissue/spheres results in a vascularized tumor-on-a-chip. Wan et al. (2021) fabricated PDMS molds with circular channels by laser cutting, and liquid gelatin was injected into the molds to obtain sacrificial gelatin templates. The sacrificial gelatin template was then placed into a chamber with cellular chambers prepared by soft lithography, collagen was added for collagen cross-linking, and the gelatin was melted to produce channels so that the microfluidic channels were embedded in the ECM. They also introduced tumor fragments obtained from MDA-MB-231 cells grown on nude mouse mammary fat pads for 8 weeks into the cell chambers and produced tumor-on-a-chip platforms with a vascular system. Hachey et al. (2021) constructed a tumor-on-a-chip platform using a similar approach to that of Wan et al. However, they introduced a mixture of normal human lung fibroblasts, endothelial cells, and tumor microtissues in the cell compartment and added laminin to facilitate the anastomosis of endothelial cells and microfluidic channels. On the seventh day, they successfully observed anastomosing tumor vascular networks in the cellular compartment using fluorescence microscopy. Nashimoto et al. (2020) used soft lithography to construct a microfluidic device with three channels. They placed tumor spheres encapsulated in a fibrin-collagen gel in the central channel and introduced endothelial growth medium and endothelial cells to form blood vessels in the channels on either side. Soft lithography enables precise design of microfluidic systems, and vascularized tumor-on-a-chip platforms can be prepared by introducing different cells into delineated cellular compartments and microfluidic channels.

### Hydrogel molding using needles

Briefly, this method is intended to create a single microfluidic microchannel by inserting or embedding a cylindrical object (such as a microneedle, wire, or capillary tube) into a hydrogel matrix; after the hydrogel has been fully crosslinked, the object is gently removed. After this step, a tubular space is formed. Many studies have demonstrated that the inner surface of the microchannel can be lined with epithelial cells, forming a confluent cell monolayer for vascularization (Nguyen et al., 2013; Wong et al., 2013). Stainless steel needles are widely used as the template. Vascularized tumor-on-a-chip platforms are prepared by encapsulating tumor cells and their stromal cells in the hydrogel inserted by the needle, or by seeding tumor spheres in the channels formed by the needle insertion.

Kim et al. (2022) inserted three needles of different diameters into their device, added a hydrogel composed of type I collagen and fibronectin, and removed the needles after gelation to create hollow channels inside the hydrogel. They seeded endothelial cells in the central channel, tumor cells on one side, and no cells on the other side to observe the effect of tumor cells on angiogenesis. By adjusting the volume difference between the hollow channel and the tumor channel, they successfully controlled the direction of interstitial flow within the hydrogel. This method makes the preparation of cellular compartments and microfluidic channels very easy. By adjusting the diameter of the needles, it is also possible to change the direction of interstitial flow, a very important parameter in tumor growth. However, it is difficult to simulate the spatial orientation of blood vessels using microneedles as templates. Miller et al. (2018) encapsulated cancer cells in collagen gel, withdrew mandrel rods to form hollow channels after complete gelation, and seeded endothelial cells in the channels to form blood vessels. Researchers have also developed a lymphoma-on-chip model with a vascularized, perfusable, round microchannel. The PDMS was molded around a stainless steel wire; then, a hole was punched in it. The hole was sealed using a diffuse large B-cell lymphoma hydrogel, which was then allowed to polymerize. After that, a plasma-bonded glass coverslip was used to seal the hydrogel. The stainless steel wire was taken out, and endothelial cells were seeded and grown in the remaining microchannel to form the vessel (Mannino et al., 2017). In both studies, the vessels prepared by needling passed through the tumor tissue, forming a tumor cell–endothelial cell interface, which was useful for observing the interaction between tumor cells and endothelial cells. With the addition of more cell types, such as tumor stromal cells wrapped in gel and immune cells delivered within the vessels, *in vitro* reproduction of the TME will become possible.

### 3D bioprinting

3D bioprinting is a technique that is widely used in many fields to fabricate sophisticated 3D structures using a layer-by-layer approach. This technique has been rapidly introduced into the field of tissue engineering. 3D bioprinting facilitates precise control of the deposition of hydrogel biomaterials to create complicated biomimetic patterns that would be impossible to create using conventional microfabrication methods (Yin et al., 2018). Through properly controlled cell and biomaterial deposition, it enables the construction of highly organized 3D vascular networks.

Extrusion bioprinting is a versatile technique that is most frequently used in 3D bioprinting. It allows customization from an engineering perspective, and a range of materials can be employed. Sacrificial inks are essential for the successful bioprinting of vascularized structures; they are printed in the matrix and liquified or washed off after cross-linking or polymerization, leaving a hollow structure inside the material (Wu et al., 2011). In general, sacrificial templated 3D bioprinting is used to form regions that should be empty in the final structure. The mechanical strength of the bioink must be sufficient to maintain a defined shape during the casting process (Albritton and Miller, 2017).

Neufeld et al. (2021) used Rhino 3D modeling software to design a vascular-like structure including bending, branching and anastomosis, which was printed using Pluronic F-127 as a sacrificial ink. The outer layer of this vascular-like structure was then wrapped with glioblastoma bioink and sealed within the microfluidic system. As the sacrificial ink was released, hollow channels were created; a mixture of endothelial and pericytes was then injected directly into the hollow channels and incubated in rotation to form blood vessels. On the fifth day, they successfully observed a vascular network using fluorescence microscopy. Instead of using a sacrificial template, Cao et al. (2019) printed a hollow pipe based on the principle that calcium chloride solution promotes immediate physical cross-linking of alginate. They designed a three-layer structure at the print tip, with the calcium chloride solution ejected from the inner and outer layers and the bioink containing the alginate component ejected from the middle layer, resulting in the precise deposition of a curved hollow tube. The vascular systems obtained by 3D printing have a more complex spatial structure compared with those generated by simple methods such as needle molding. In addition, by changing the hydrogel composition in the bioink, the permeability of blood vessels and lymphatic vessels can be individually altered during the printing process.

Below, we list the studies based on tumor-on-a-chip platforms that have been published in recent years (Table 1).

**TABLE 1:** Breakthroughs in tumor-on-a-chip technology for building vascularized cancer models.

Microfluidic system construction method	Materials for simulating blood vessels	Application or description	Highlights	References
Hydrogel molding by needles	Type I collagen and fibrin hydrogel; Human umbilical vein endothelial cells (HUVECs)	Assessment of anticancer drug and immune cell transport in a vascularized lung cancer model	Has application potential for screening of anti-cancer drugs, targeting tumors and blood vessels, or for assessing the efficacy of anti-cancer treatments	Kim et al. (2022)
Photolithographic	Collagen, gelatin gel	High-throughput 3D drug dynamic delivery system applied to anti-cancer drug screening	Drug sensitivity screening is closer to clinical significance and can test for combination drug effects	Wan et al. (2021)
3D bio-printing	Pluronic F127; Human endothelial cells; Human microvascular perivascular brain cells	Perfusable 3D bioprinted tumor model of glioblastoma	Adequately mimics brain tissue; predicts appropriate clinical treatment for each individual	Neufeld et al. (2021)
Soft lithography	Fibrinogen; NHLFs and ECFC-ECs	*In vitro* vascularized micro-tumor model of human colorectal cancer	The *in vitro* vascularized microtumor model of human colorectal cancer recapitulates tumor cell heterogeneity, vascular disruption, tumor-microenvironment interactions, and its therapeutic response	Hachey et al. (2021)
Hydrogel molding by needles	Collagen I; Fibronectin; HUVEC-GFP	Revealing the kinetics of mosaic angiogenesis in breast cancer	Real-time imaging of the cellular mechanisms of mosaic angiogenesis and vascular defect generation	Silvestri et al. (2020)
Photolithographic	Fibrin—Collagen Gel; HUVECs	Integrating perfusable vascular networks with tumor spheres to build drug screening platforms	Uncovering the impact of fluid flow within the lumen of vascular networks on the assessment of tumor activity during drug screening	Nashimoto et al. (2020)
Soft lithography	Collagen I; Human dermal microvascular blood endothelial cells	Triple-negative breast cancer (TNBC) metastasis in common metastatic organs of the bone and lung	Parenchymal cells determine the selective extravasation of TNBC cells	Kwak and Lee, (2020)
Photolithographic	Fibrin gels; ECFC-EC and NHLF	Summarize important features of the tumor immune and vascular microenvironment	Trying to understand the mechanisms of the immune response in tumor progression	Bi et al. (2020)
Soft lithography	GelMA Hydrogel; HUVECs	Effect of cancer cell-monocyte interactions on T cell recruitment	Understanding how the cancer microenvironment is shaped to create the conditions for immunotherapy development	Aung et al. (2020)
3D bio-printing	BdECM gels; HUVECs	Recapitulation of clinically observed patient-specific resistance to concurrent radiotherapy and temozolomide treatment	Identifying Glioblastoma Patient-Specific Drug Sensitivities to Help Develop Tumor-Personalized Treatments	Yi et al. (2019)
Photolithographic	Fibrin gels; GFP-HUVECs	Studying the dynamics of brain tumor stem cell-like cells (BTSC) in the perivascular ecological niche (PVN)	Outline *in vivo* tumor cell dynamics and heterogeneity and provide new ways to study patient-specific tumor cell function	Xiao et al. (2019)
Hydrogel molding by needles	Collagen I; Fibronectin; HUVEC	Visualization and quantitative analysis of endothelial and individual tumor cell responses to chemotherapy	Helps improve the design of new drug delivery systems and guide clinical dosing regimens	Wong et al. (2019)
Hydrogel molding by needles	Fibrin gels; HUVECs	Simulating vascular invasion and tumor-vessel interactions in pancreatic ductal adenocarcinoma	To investigate PDAC-driven endothelial ablation processes and mechanisms of tumor vascular decompensation	Nguyen et al. (2019)
Photolithographic	Matrigel solution; Human colonic microvascular endothelial cells (HCoMECs)	Simulating the human colorectal tumor microenvironment and evaluating precise nanodrug delivery	Provide the possibility of precision nanomedicines for personalized therapeutic research	Carvalho et al. (2019)
3D bio-printing	GelMA, alginate and PEGDA or PEGOA	Constructed an *in vitro* tumor model containing bioprinted blood/lymphatic vessel pairs	Simulated biomolecular and drug transport kinetics to provide a platform for future drug development	Cao et al. (2019)
Photolithographic	Fibrin gels; ECFC-EC and NHLF	Establishment of an *in vitro* model of breast cancer that simulates biological mass transfer near the end of capillary arteries	Simulating the physiological delivery of drugs to tumors through vascular networks to assess efficacy	Shirure et al. (2018)
Photolithographic and soft lithography	Fibrin gels; HUVECs	To study the maturation of breast cancer cell invasion and endocytosis and the vascular system in the impact of tumor-vascular crosstalk	Offers the possibility to explore the metastatic cascade response and develop efficient cancer therapies	Nagaraju et al. (2018)
Hydrogel molding by needles	Collagen gels; HUVECs	Observation of angiogenic germination stimulation in primary human clear cell renal cell carcinoma (ccRCC)	Studying tumor-vascular cell interactions and developing novel and personalized anti-tumor therapies	Miller et al. (2018)
Self-assemble	Decellularized 3D bone matrix; EC with MSCs	Construction of perivascular ecotone to study bone metastasis colonization in breast cancer	Facilitates the development of drugs that target bone metastatic tumor cells and help manage the risk of metastatic recurrence	Marturano-Kruik et al. (2018)
Soft lithography	Collagen gel	Investigating the immunosuppressive potential of monocytes against HBV-specific TCR T cells and the role of PD-L1/PD-1 signaling	Predicting the efficacy of preclinical TCR T cells.; Improving Current Immunotherapy Strategies	Lee et al. (2018)
Soft lithography	Collagen Hydrogel; C166-GFP EC	Studying immune-vascular and cell-matrix interactions in glioblastoma (GBM)	Emphasizing the importance of macrophage-associated immunosuppression in GBM angiogenesis; Screening for novel anti-angiogenic therapies	Cui et al. (2018)
Hydrogel molding by needles	Collagen I; HUVECs	Demonstrate that human breast cancer cells are able to disrupt the vascular endothelium by cell division and separate into the circulation	Provide a workable engineering platform for studying the endocytosis of tumor cells on intact endothelium	Wong and Searson, (2017)
Hydrogel molding by needles	Fibrin and Collagen I; HUVECs	Investigate the effects of retinoid therapy on the neuroblastoma vasculature and drug resistance	SOX2 was proposed as a possible therapeutic target for neuroblastoma	Villasante et al. (2017)
Soft lithography	Fibronectin and Gelatin; Primary human breast tumor-associated endothelial cells (HBTAEC)	*In vitro* tumor-endothelial interaction model of breast cancer was constructed	Possible platform for studying cell-cell/cell-drug carrier interactions and rapid screening of cancer drug treatments/vectors	Tang et al. (2017)
Photolithographic and soft lithography	Fibrin gels; ECFC-EC and NHLF	Created an array vascularized microtumor platform for drug screening	Large-scale drug screening of multiple tissues can be performed simultaneously	Phan et al. (2017)
Hydrogel molding by needles	Fibronectin and Arginine sulfated acyl glycine (RGD-SH); Mouse lung microvascular endothelial cells (MLMVECs)	A microfluidic platform that accurately summarizes the tumor microenvironment of diffuse large B-cell lymphoma (DLBCL)	Contributes to microenvironmental studies and drug delivery and design in diffuse large B-cell lymphoma (DLBCL) tumors	Mannino et al. (2017)
Photolithographic	Collagen gels; Human dermal lymphatic microvascular endothelial cells (HMVEC-dLyAd)	Reconstruction of tumor cell interactions with peritumor cells and non-cellular components	Contributes to the in-depth study of the pathophysiology of the tumor microenvironment (TME)	Chung et al. (2017)
Photolithographic	HUVECs	A multi-organ microfluidic chip was constructed to mimic lung cancer metastasis	Created a replicable model of lung cancer growth, invasion and metastasis	Xu et al. (2016)
Soft lithography	Fibrin gels; ECFC-EC	Assessing the therapeutic response of colorectal and breast cancer cells to vascular targeting agents	Provides a model for *in vitro* studies of vascularized solid tumors	Sobrino et al. (2016)
Soft lithography	Fibrin gels; Endothelial cells (ECs), mesenchymal stem cells and osteoblastic differentiated cells (OBs)	Study of extravasation of breast cancer cells in vascular networks formed by co-culture of endothelial and hBM-MSC-derived mural cells	Constructed a vascular network platform different from the microvascular network made of endothelial cells only	Jeon et al. (2015)
Photolithographic	Fibrinogen; HUVECs and Lung fibroblasts (LFs)	*In vitro* reproduction of the EC-cancer interface to observe angiogenic responses as well as tumor cell infiltration and response to anti-vascular drugs	Provides the possibility to study tumor transendothelial cell migration and screening of antitumor drugs	Lee et al. (2014)

## Tumor metastasis research using tumor-on-a-chip platform

More than 90% of cancer-related deaths are due to subsequent disease caused by tumor metastasis (Seyfried and Huysentruyt, 2013). Tumors can spread through blood vessels and lymphatic vessels, resulting in metastasis through a series of metastatic cascade reactions (Ganesh and Massagué, 2021). Cancer cells are isolated from the primary tumor, infiltrating the vascular system (with or without passing through lymphatic vessels and lymph nodes) and are then transported to the target organ, where they extravasate, seed, and survive (Labelle and Hynes, 2012). Therefore, models with infusible vasculature and even lymphatic vessels can best simulate this process and help to improve our understanding of cancer metastasis. Although several 3D cancer models, including spheroids (Tanaka et al., 2018; Courau et al., 2019) and organoids (Tsai et al., 2018; Jacob et al., 2020), have been able to reproduce the 3D structure of tumor tissue to a great extent, they lack the capacity for detection of tumors in a dynamic microenvironment (Verjans et al., 2018). By contrast, using a tumor-on-a-chip platform enables the creation of infusible vessels owing to the combination of microfluidic systems with 3D models.

### Angiogenesis and metastasis

Microfluidics has made it possible to generate vascular–tumor interfaces *in vitro* (Lee et al., 2014), and this has led to a number of studies on the process of tumor metastasis. Chung et al. (2017) established a microfluidic platform to simulate both angiogenesis and lymphatic angiogenesis in the TME. They observed both lymphatic and vascular buds wrapped around colonies of tumor cells at the end of the bud, forming tight contacts that may be involved in determining biological behaviors of the tumor such as metastasis and immune escape.

Sobrino et al. (2016) inoculated two external microfluidic channels with endothelial cells to simulate small arteries (high pressure) and small veins (low pressure) in tumor tissue by controlling the pressure of the channels. They introduced tumor cells and vascular endothelial cells together into the cell chamber in a dynamic environment and compared them with vascular endothelial cells when cultured alone to observe tumor–endothelial cell interactions. Following inoculation with tumor cells of different types, significant differences in angiogenesis and tumor growth rates were observed, despite the fact that all tumor spheroids exhibited tight apposition to the microvascular network. This may have been due to the unique remodeling of the microenvironment by different tumor cells.

To accomplish metastasis, tumor tissue rapidly develops immature vascular networks, and these poorly functioning vessels can increase the risk of metastatic dissemination (Viallard and Larrivée, 2017). To explore how tumor cells enter the circulation through these vascular networks is fundamental to the in-depth study of tumor metastasis. Tang et al. (2017) seeded primary human breast tumor associated endothelial cells into vascular compartments to establish an in-vivo-like microvascular network with intact vascular endothelial lumen, and seeded MDA-MB-231 tumor cells in tumor compartments surrounded by the microvascular network. They observed that the presence of MDA-MB-231 tumor cells increased vascular permeability by impairing endothelial cell–cell junctions. Nguyen and coworkers observed that pancreatic ductal carcinoma (PDAC) tumor cells were able to rapidly invade blood vessels, replace endothelium, and occupy the lumen, a phenomenon that may underlie the rapid metastasis of tumors (Nguyen et al., 2019). They constructed a system consisting of a mimic conduit channel for PDAC cells and a perfused vessel composed of endothelium in parallel. They added transforming growth factor (TGF)-β receptor inhibitors by microfluidic techniques. Over 7 days of treatment, a significant reduction in ablation of vascular endothelial cells was observed compared with the case without the addition of inhibitors. In static patterned culture, they demonstrated that endothelial ablation of PDAC was mediated by the activin–ALK7 pathway *via* effects on the TGF-β receptor signaling pathway.

Certain tumor types have been found to prefer certain organ microenvironments when metastasizing; this phenomenon is referred to as organ-specific cancer metastasis (Hoshino et al., 2015). Therefore, when developing models for metastatic studies, it is important to include specific organs or tissues where tumors are more inclined to metastasize.

Based on organ-on-a-chip technology, Xu et al. (2016) constructed an *in vivo* metastasis model of lung cancer. They co-cultured bronchial epithelial cells and stromal cells between microvascular channels and the air interface to form a lung-on-a-chip that could simulate physiological respiratory movements. After co-culturing the lung cancer cell lines on the lung-on-a-chip, a syringe pump was used to simulate blood circulation, allowing culture fluid to be delivered from the lung cancer chip to the downstream organ-on-a-chip through the microvascular channel connecting the cell chambers. Astrocytes, osteoblasts, and hepatocytes were cultured in down-flow cell culture chambers to mimic brain, bone, and liver, respectively, three organs where lung cancer often metastasizes (Riihimäki et al., 2014). The researchers successfully demonstrated that this platform could simulate the invasion and metastasis of lung cancer *in vivo* and that more studies could be conducted on this platform owing to its reproducibility.

Another vascularized tumor model for organ-specific cancer metastasis was fabricated by Kwak and Lee (2020). They engineered blood vessels by inoculating human microvascular blood endothelial cells and inoculated target parenchymal organ cells in the gel surrounding the engineered vessels to mimic the ECM of the target organs; then, they inoculated breast cancer cells with specific metastatic properties into the engineered vessels to study the extravasation of breast tumors in distant organs, including bone and lung. They found that osteoblasts played a key part in the selective extravasation of bone MDA-231, an MDA-MB-231 derivative with a tendency to bone metastasis. These models demonstrate the role of vascularized tumor-on-a-chip technology in providing reproducible models of metastasis and extravasation of tumors in different organ microenvironments.

### Tumor-associated macrophages: Involvement in metastasis

Many studies support the idea that tumor innate immune cells have important roles in tumor invasion and metastasis (Güç and Pollard, 2021). Among these cell types, one of the most widely studied is tumor-associated macrophages (TAMs), which have been shown to assist tumor cells in lymphatic and hematologic metastasis (Weichand et al., 2017; Wang et al., 2018). However, most of these studies have been based on mouse tumor models or human samples, whereas the application of microfluidic models provides the opportunity to observe the interaction between TAMs and tumor cells and stroma in a dynamic TME.

Bai et al. (2015) co-cultured tumor cell aggregates with multiple subtypes of macrophages and cultured endothelial monolayers in parallel adjacent compartments to mimic capillaries. They observed that M2a macrophages contributed to the infiltration and release of tumor cells from the cancer cell aggregates, which may represent the beginning of tumor metastasis. Cui et al. (2018) made two parallel channels in a hydrogel containing macrophages, inoculated them with endothelial cells for vascularization, and inoculated glioblastoma-like tumors (GL261 or CT-2A) and macrophages in a perivascular hydrogel. They modulated biomolecules in the TME by altering attachment molecules on type I collagen monomers in hydrogels and thereby confirmed that integrin αvβ3 plays an important part in endothelial cell–macrophage interactions and ultimately affects inflammation-driven angiogenesis.

TAMs have been shown to interact with tumor cells and endothelial cells, possibly leading to the dissociation of tumor cells and angiogenesis, which is often a prerequisite for metastasis to occur.

Bi and coworkers established a tumor microfluidic platform by inoculating a microvascular system with human lung fibroblasts and human endothelial colony-forming cell-derived endothelial cells to form simulated blood vessels and introducing a 1:1 ratio of mixed tumor cells and immune cells (induced to differentiate to M1 or M2 macrophages, respectively) into the side chambers (Bi et al., 2020). Angiogenesis and tumor volume in the central chamber, which was connected to the side chamber by a microvascular network, were observed in the presence of different TAMs. In the presence of M1 macrophages, tumor growth was significantly reduced, angiogenesis was completely blocked, and tumor migration/invasion was inhibited. However, in the presence of M2 macrophages, tumor growth and angiogenesis were not affected, whereas tumor migration to the central compartment was significantly increased. This demonstrates the direct involvement of different subtypes of macrophages in regulating tumor angiogenesis and metastasis.

In solid tumors, vascular function is often abnormal, and focal hyperpermeability of the vessels can lead to leakage of plasma from the vessels into the interstitial space of the tumor; this is called interstitial fluid (IF). The extravascular hydrostatic pressure due to interstitial fluid accumulation is called interstitial pressure (Lunt et al., 2008). Elevation of IF pressure has been shown to contribute to cancer progression and poor patient survival (Hompland et al., 2012), and there is evidence that IF can cause macrophage chemotaxis and tumor cell metastasis (Rutkowski and Swartz, 2007).

Lee et al. (2020) constructed a migration model of TAM by microfluidics, in which they cultured tumor cells and macrophages in two parallel channels and controlled the flow of IF between the two channels at a constant rate within the range of normal flow rates in tumor tissue. They observed that IF-exposed macrophages migrated faster and were more directional than untreated controls, whereas IF-pretreated macrophages resulted in enhanced cancer cell invasiveness when co-cultured with cancer cells (Li et al., 2018b). Therefore, these findings confirmed that IF promotes cancer aggressiveness by enhancing macrophage migration.

**FIGURE 3:**
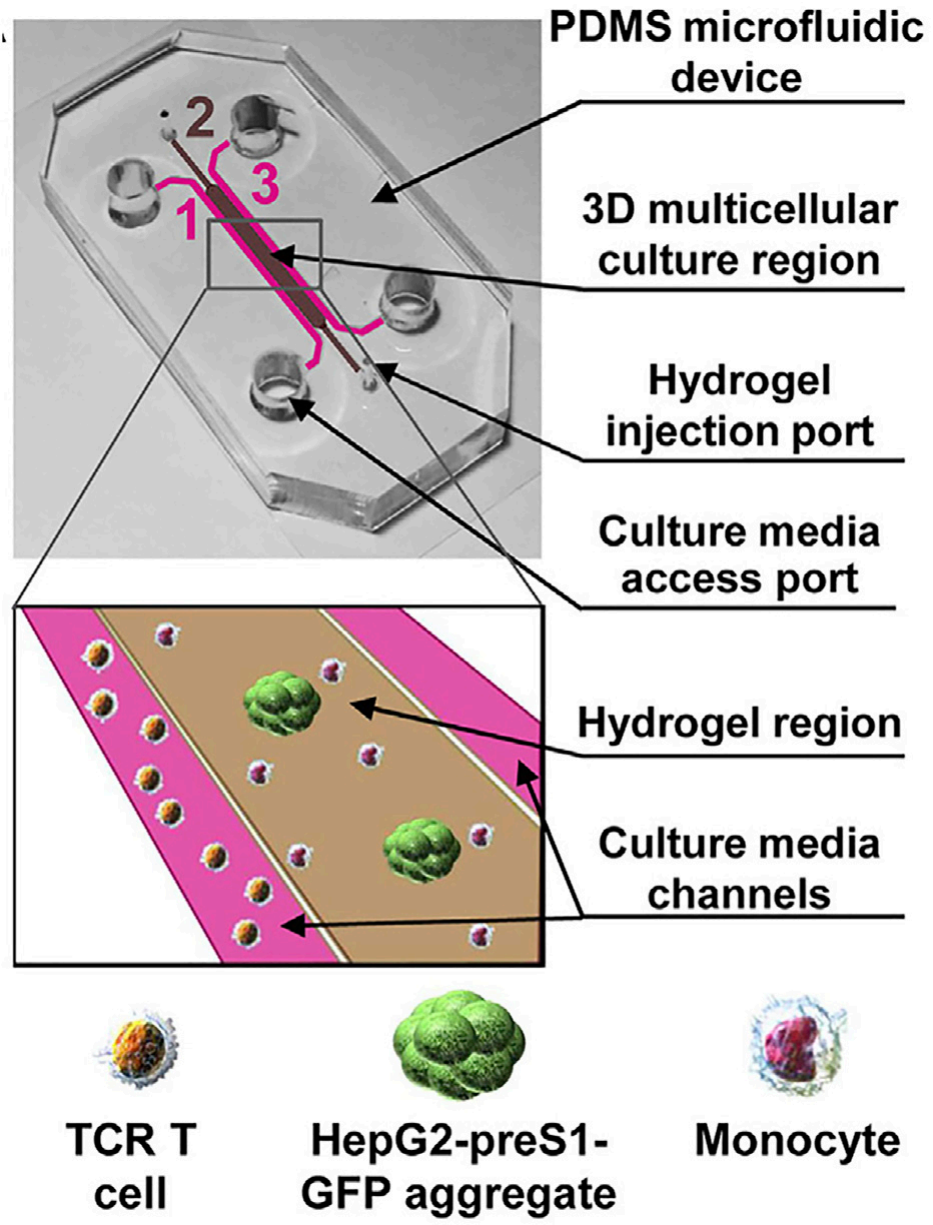
Human monocytes were cultured in collagen gels together with the labeled human HCC cell line HepG2 (HepG2-preS1-GFP), while hepatitis B virus (HBV)-specific TCR T cells passed through the tumor aggregation zone from both sides of the fluid channel to mimic the dynamic microenvironment of hepatitis B virus (HBV)-related hepatocellular carcinoma. Adapted under the terms of the Creative Commons Attribution License (CC BY). (https://creativecommons.org/licenses/by/4.0/). Copyright: Lee et al. (2018), Adriani, Ceccarello, Pavesi, Tan, Bertoletti, Kamm and Wong.

Tumor metastasis requires evasion of immune system surveillance, and TAM have been shown to be important regulators of tumor-immune system interactions (Farajzadeh Valilou et al., 2018). Monocytes/macrophages have been shown to significantly regulate effector T cell activity *via* PD-L1 (Cai et al., 2019). Studies targeting monocyte immunosuppression have been performed. For instance, (Lee et al., 2018), co-cultured monocytes with tumor cell aggregates to observe their toxic effects on T cell receptor-transduced T cells in microfluidic channels on both sides (Figure 3). Their results demonstrated that monocytes could affect retroviral transduction through a PD-L1/PD-1-dependent mechanism but did not affect mRNA electroporation T cell receptor-redirected T cell cytotoxicity. Another experiment demonstrated that the presence of monocytes increased the recruitment of T cells at tumor sites (Aung et al., 2020).

Taken together, these results indicate that TAMs are involved in almost every step of the tumor metastasis cascade reaction and have important roles in the process of tumor metastasis. Studies on the interactions of TAMs with the TME will provide more possibilities for deepening our understanding of the TME and improving immune drug development.

## Anticancer drug discovery using tumor-on-a-chip platform

2D models are often adhered to plastic substrates for culture, such that it is difficult to represent the complex physiological 3D environment, and drug trials that ignore *in vivo* structural effects may lead to discredited conclusions (Antoni et al., 2015). Besides the effects of the TME, blood circulation and lymphatic drainage may affect the distribution and action of drug molecules in the tumor tissue. The drug enters the vasculature and reaches the tumor site through the blood circulation, passing through the vessel wall into the tumor tissue by various means (diffusion or active transport), while excess drug eventually flows into the lymphatic system. Previous methods of incorporating vascular networks into tumor models have tended to co-culture endothelial cells inside and outside tumor spheres, but the vascular networks thus formed are mostly non-infusible (Klimkiewicz et al., 2017; Shoval et al., 2017). Existing drug studies on tumor models constructed using tumor-on-a-chip technology have shown that perfusable vascular networks contribute to a more realistic evaluation of the efficacy of existing anticancer drugs.

Work by Nashimoto et al. (2020) showed that the volume of tumor spheroids decreased with increasing concentration of paclitaxel administered in static culture with or without a vascular network, but the reduction in tumor spheroid volume was greater in cases with a vascular system. However, when the drug was administered by perfusion, tumor volume did not decrease in a drug-dependent manner, as seen under static culture. The volume of spheroids with a vascular system was greater than the volume of spheroids without a vascular system at 5 ng/ml treatment under perfusion conditions, possibly because cell proliferation caused by nutrients delivered by perfusable vessels exceeded the cell death caused by paclitaxel. The same results were observed by (Hachey et al., 2021), who cultured 2 cell lines, HCT116 and SW480, in 2D culture and in a vascularized tumor model, respectively, and observed that in the 2D cultures the tumors grew rapidly and were very sensitive to drug treatment. However, in the vascularized model, the growth of both cell lines was much slower, and the drugs retarded but did not reverse tumor growth.

In addition to the role of infusible vessels in bringing nutrients that promote tumor cell proliferation to the tumor site, the possibility that the circulatory system may carry away drugs and thus reduce their concentrations may contribute to reduced drug efficacy. Cao et al. (2019) constructed a tumor-on-a-chip system with a pair of infusible empty vasculature and single-outlet lymphatics by bioprinting and achieved different permeabilities for blood vessels and lymphatics by regulating the content of PEG molecules with straight-chain and branched structures in the bioink. They demonstrated that under treatment with doxorubicin, the model where both blood vessels and lymphatic vessels were present showed higher cell viability than the model where only blood vessels were present. This may have been because the presence of a lymphatic drainage system led to rapid clearance of the drug, whereas the drug tended to accumulate in the presence of a single vessel. Moreover, because increasing the number of branched chains of PEG can improve hydrogel permeability (Jia et al., 2016), this system could better simulate tumor vasculature with different permeabilities by changing the bioink composition. However, neither the blood vessels nor the lymphatic tracts in this experiment contained cells, which could have led to a lack of active transport of the drug and thus experimental error.

Although all of the above studies suggest that the presence of infusible vascular networks in tumor models may have an impact on drug distribution and concentration, the relationship between drug distribution and perfusion volume has still not been clearly demonstrated. Carvalho and coworkers successfully developed a colorectal tumor-on-a-chip model for accurate assessment of nanodrug delivery (Carvalho et al., 2019). They attached human colonic microvascular endothelial cells to microfluidic channels to form a microvascular system and achieved good control of drug molecule gradients in the solution of the vascular system by microfluidics. However, in this model, they only observed the formation of vascular buds; no anastomosis was formed between endothelial cells. They selected CMCht/PAMAM dendrimer nanoparticles as drug-loaded nanoparticles in order to achieve a controlled and stable drug gradient. These particles are capable of being modified by fluorescent molecules such as fluorescein isothiocyanate and can function as cell trackers (Carvalho et al., 2017). With these particles, they were able to visualize the drug delivery range and potentially establish a standard for drug efficacy comparison, for instance, by controlling the flow rate and the nanoparticles for drug release and observing the efficacy. This may also be the way forward for accurate assessment of drug efficacy using tumor-on-a-chip platforms.

The tumor-on-a-chip is also an alternative platform for efficient drug screening. Wan and coworkers developed a 3D ECM model consisting of collagen type I fibers forming a scaffold with embedded microfluidic channels (Wan et al., 2021). They achieved simultaneous embedding of multiple tumor tissues at the same distance from the microfluidic channels and tested the therapeutic response of five mouse xenografts derived from the MDA-MB-231 human breast cancer cell line to single and combination drugs by perfusion administration. This approach, which mimics the *in vivo* tumor vascular system for drug screening, can significantly reduce costs and allows for parallel experiments. However, true endothelial vasculature is not present in this system.

In personalized medicine, preserving the original drug sensitivity of the tumor also has important implications in drug screening (Yi et al., 2019). used 3D printing to create vascularized models with spatial anatomical similarities to glioblastoma; they successfully simulated pathological features and accurately reproduced the treatment resistance of patients. Hachey et al. (2021) constructed a dynamic 3D tumor model using tumor-on-a-chip technology. They cultured HCT116 colorectal cancer cells in a 2D pattern (monolayer) culture, a vascularized model culture, and an allogeneic culture, which they used to perform transcriptomic analysis of 770 cancer-related genes. They found that the gene expression patterns of tumor cells from the vascularized tumor model closely matched those of *in vivo* tumor origin, and there was minimal variability between replicate cultures, highlighting the robustness and reproducibility of the vascularized tumor model. Moreover, tumor cell heterogeneity was better preserved in the dynamic 3D TME. The dynamic 3D TME retained tumor heterogeneity extremely well and could thus be used to localize tumor resistance genes, clarify tumor resistance mechanisms, and develop targeted drugs.

## Applications in common tumor models

Tumor-on-a-chip technology offers the possibility of constructing perfusable vascularized tumor models. The microenvironments of different types of tumors vary greatly; here, we list a few scenarios in which this technology might be applied to pancreatic cancer, brain tumors, and hematological malignancies. An attempt is made to depict the potential applications of tumor-on-a-chip technology in constructing models of highly aggressive tumors, establishing the unique microenvironment of brain tumors, and constructing nonsolid tumor models.

### Tumor-on-a-chip for pancreatic cancer

PDAC is a highly aggressive disease that is associated with overexpression of angiogenic growth factors and their receptors (Korc, 2003), but tumor masses obtained from patients often show reduced or absent vascularity (Craven et al., 2016). Thus, angiogenesis in PDAC masses is necessarily uniquely regulated by tumor cells, allowing microvessels to provide access to the circulation for tumor cells at an early stage, while vascular reduction occurs in the later stages of the tumor and restricts the ability of chemotherapeutic agents to reach the tumor site.

Nguyen et al. (2019) constructed a system consisting of a bionic ductal channel of PDAC cells and perfused vessels composed of endothelium in parallel. They observed that under fetal bovine serum stimulation, PDAC cells spread along the long axis of the vessel in a winding pattern, invading and removing the endothelium and the collagen IV layer deposited by endothelial cells. They observed the same phenomenon in a genetically engineered mouse model of PDAC, where PDAC invaded the vasculature and eventually replaced the endothelium, leaving behind luminal structures lined by tumor cells and demonstrating that endothelial ablation occurs *in vivo*.

Another experiment incorporated the lymphatic system into the development of a metastatic model of pancreatic tumors. Nishiguchi et al. (2018) seeded the pancreatic cancer cell line BxPC3 from the pancreas into a capillary and lymphatic vessel model formed by co-culture of HUVECs, normal human dermal fibroblasts, and human lymphatic endothelial cells (LECs) to observe invasion and extravasation. They found that invasion of BxPC3 cells selectively induced apoptosis of HUVECs only. The invasion of cancer cells into lymphatic vessels did not cause apoptosis of LECs. Showing random movement in the tubular structures, cancer cells eventually left the lumen and exfiltrated. The interactions of capillaries and lymphatic vessels with cancer cells demonstrated by these experiments indicate the possibility of exploring a range of tumor behaviors in pancreatic metastasis.

### Tumor-on-a-chip for brain cancer

The reconstruction of the TME in brain is more challenging than in other cancers owing to the unique nature of the brain (Quail and Joyce, 2017). Here we present some examples of brain tumor models constructed by applying tumor-on-a-chip technology, which have led to breakthroughs in simulating the unique microenvironment of brain tumors.

Glioblastoma is the most common and incurable primary malignant brain tumor (Omuro and DeAngelis, 2013). Glioblastoma stem cells are believed to be present in tumors and capable of continuous differentiation, making the tumors highly resistant to treatment (Gilbertson and Rich, 2007). Glioblastoma stem cells preferentially accumulate in areas near the microvasculature, which is called the perivascular ecological niche in brain tumors (Aderetti et al., 2018).

**FIGURE 4:**
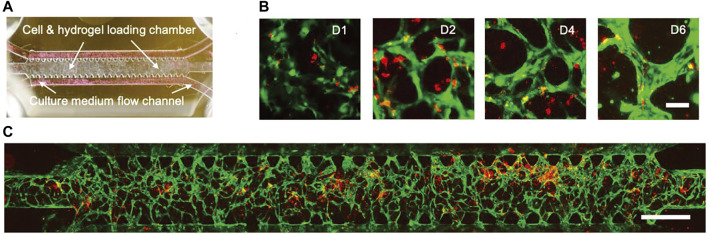
Perfusable vascularized glioblastoma model for dynamic localization and live cell imaging of individual brain tumor cells near microvessels. **(A)** Microfluidic device containing a cell/gel loading microchamber and media flow channels on both sides; **(B,C)** Microvessel formation and loading of individual BTSCs. Green: GFP-HUVECs. Red: BTSCs. Adapted under the terms of the Creative Commons Attribution License (CC BY). (https://creativecommons.org/licenses/by/4.0/). (Xiao et al., 2019) Copyright 2019 The Authors. Published by WILEY-VCH Verlag GmbH and Co., KGaA, Weinheim.

Xiao et al. (2019) successfully established a perfusable vascularized glioblastoma model and achieved dynamic localization and live-cell imaging of individual brain tumor cells in the vicinity of microvessels to investigate the interactions of individual tumor cells and microvessels (Figure 4). They observed that brain tumor stem-cell-like cells preferentially localized to the perivascular area and exhibited either quiescent or aggressive behavior. This model could well describe the interaction between tumor cells and microvasculature, as well as the movement and morphology of individual tumor cells, and has great promise for many applications such as tracking of tumor cell migration.

Despite the high vascularity of neuroblastoma, some drugs that have shown anti-angiogenic effects in preclinical neuroblastoma models, such as bevacizumab, have not been found to have a positive effect in clinical trials (Modak and Cheung, 2010). This may be related to the mechanism of angiogenesis. The only currently described mechanism of angiogenesis in neuroblastoma is angiogenic mimicry, in which the tumor cells acquire endothelial cell properties and form tubular structures (Pinto et al., 2016). Therefore, it is important to establish a model of vascularized neuroblastoma to observe the mechanism of angiogenesis and to test the effect of anti-vascular drugs.

Villasante et al. (2017) cultured prevascularized triple-layered neuroblasts in a vascular bed formed by HUVECs and observed the angiogenesis and drug response of prevascularized neuroblasts under perfusion. The results demonstrated that the vasculature in the cell sheets originated from HUVECs and neuroblastoma cells that transdifferentiated into tumor-derived endothelial cells. Treatment with isotretinoin in this perfusable vascularization model of neuroblastoma was observed to induce apoptosis and reduce total CD31 expression in neuroblastoma cells to cause disintegration of the vascular network, but it did not affect tumor-derived endothelial cells or vascular-like structures.

### Tumor-on-a-chip for hematological malignancies

Hematological malignancies include various forms of leukemia, lymphoma, and myeloma (Lichtman, 2008). In the bone marrow (BM) microenvironment, hematopoietic stem cells are tightly regulated to maintain the population and to produce mature immune and hematopoietic cells (Clara-Trujillo et al., 2020). Alterations in the BM niche (BMN) may cause malignant mutations (Walkley et al., 2007), and remodeling of the BNM by malignant cells in turn may further promote tumor development (Arranz et al., 2014; Hawkins et al., 2016).

Studies have shown that the BMN is associated with tumor development and drug resistance (Méndez-Ferrer et al., 2020). Therefore, reconstructing the BM microenvironment is of great importance in studying the behavior of blood cancers, and various BM models have now been proposed (Bourgine et al., 2018). BM models that use microfluidics to simulate the complexity of the BMN are known as BM-on-a-chip platforms.

Although there are many tissue-engineered BM models, blood cancer studies have mostly been performed on animal-derived BMNs (Ibraheem et al., 2019). Some studies have used polymeric materials to make porous scaffolds to construct 3D blood cancer models, but there is still a lack of BM-based tissue-engineered blood cancer models.

de la Puente et al. (2015) used fibrin gels to form 3D tissue-engineered BM cultures and construct multiple myeloma models, outlined the interactions of multiple myeloma cells with the microenvironment, and investigated changes in oxygen gradients and tumor cell drug resistance in the models. Although they observed changes in oxygen gradients and increases in angiogenic factors, definitive evidence of angiogenesis is still lacking. Li et al. (2018a) constructed a 3D leukemia model using decellularized Wharton’s jelly matrix as a scaffold material and investigated growth patterns, stem cell characteristics, and response to drugs in three human leukemia cell lines. However, this model also lacked consideration of how blood vessels are formed. These 3D models do not take into account the importance of functional vasculature in blood cancer models. In a study of breast cancer bone metastasis, Marturano-Kruik et al. (2018) constructed a human bone perivascular niche-on-a-chip and demonstrated that perivascular ecological niches in bone may promote tumor metastasis and drug resistance development. Simulating the generation of perfusable blood vessels may be a direction leading to optimization of 3D models of blood cancers.

## Outlook

Like all emerging technologies, there are unresolved issues with the tumor-on-a-chip platforms. Here we list the challenges and opportunities for oncology-on-a-chip platforms, hoping to contribute to the development of this field.

### Material optimization and scale up

Polydimethylsiloxane (PDMS) is widely used in the manufacture of tumor-on-a-chip platforms, but due to its hydrophobic nature, it tends to adsorb most anticancer drugs, which may lead to erroneous experimental conclusions and limit its clinical applications (van Meer et al., 2017). This calls for the emergence of new materials. To be more bionic, tumor-on-a-chip platforms are becoming increasingly complex. Different studies may introduce different types (e.g., stromal cells, immune cells, etc.) as well as different sources of cells when reconstructing TME *in vitro*. This leads to a decrease in operability and reproducibility. Also, the standards and guidelines for each chip are not uniform. This may encounter difficulties in large-scale experiments. For example, the lack of uniform standards for drug studies makes it difficult to achieve cross-sectional comparisons, resulting in some information being wasted. For better clinical applications, the preparation of tumor-on-a-chip platforms will seek to standardize and scale up.

### Better vascular simulation

In terms of vascular morphology and composition, the spatial relationship between tumor tissue and blood vessels cannot be easily and completely simulated. Whether tumor vascular endothelium or normal endothelial cells are used to form the vascular wall, and whether there is a meaningful difference between the two is also a question worth considering. Meanwhile, in simulating vascular function, the culture fluid is difficult to simulate real blood, and the permeability of model vessels to drugs may be different from that of real vessels. 3D printing technology has shown great potential for complex vascular configurations, and the use of 3D printing to design and fabricate tumor-on-a-chip platforms may help in addressing vascular structural mimicry. The functionality of model vessels will be further secured through the development of body fluid mimics, the creation of more complete vascular systems (e.g., the introduction of the lymphatic system), and the incorporation of tumor perfusion cycles.

### More personalized designs

With the development of precision medicine, there is a greater interest in the preparation of *in vitro* tumor models with patient specificity. It has been demonstrated that tumor-on-a-chip platforms preserve tumor heterogeneity (Hachey et al., 2021) and specificity (Yi et al., 2019) to a greater extent than conventional 3D tumor models. This all suggests that it is feasible to dynamically grow 3D tumor models with patient specificity *in vitro* using a tumor-on-a-chip platform. Also, this platform enables prediction of patient tumor progression, regression, and drug sensitivity. When combined with rapid synthetic technologies such as 3D printing, the creation of bedside tumor models will become possible in the future. The development of tumor treatment plans will be easier, more accurate and personalized. However, commercial immortalized tumor cell lines are still the more commonly used cells. At the same time, obtaining a good correlation between *in vitro* results obtained by tumor-on-a-chip platforms and clinical *in vivo* responses remains challenging. This may be since multiple organs are involved in the response *in vivo*, and how to achieve better clinical correlation is also an issue that needs to be addressed.

## Conclusion

Vascularized tumor-on-a-chip models have shown great potential for applications in the study of tumor angiogenesis, metastasis, and drug development owing to their ability to dynamically mimic the TME. Studies have been performed to clearly reproduce the spatial relationship between nascent lymphatic vessels and blood vessels in tumor tissues. Localization of individual tumor cells, as well as reproduction of tumor cells entering and leaving lymphatic vessels and blood vessels, has been achieved on tumor-on-a-chip platforms. There have also been many advances regarding drug diffusion and efficacy assessment in tumor tissues based on tumor-on-a-chip platforms. With the further imitation of tumor microenvironment and the realization of large-scale manufacturing, vascularized tumor-on-a-chip platforms will make greater contributions in the fields of drug screening and precision medicine.
